# Washing Machine Dynamic Model to Prevent Tub Collision during Transient State

**DOI:** 10.3390/s20226636

**Published:** 2020-11-19

**Authors:** Beatriz Sánchez-Tabuenca, Carmen Galé, Juan Lladó, Cristian Albero, Roberto Latre

**Affiliations:** 1 Mechanical Engineering Department, University of Zaragoza, María de Luna 3, 50018 Zaragoza, Spain; juan.llado@unizar.es (J.L.); calbero@unizar.es (C.A.); 2 Statistical Methods Department, IUMA, University of Zaragoza, María de Luna 3, 50018 Zaragoza, Spain; cgale@unizar.es; 3 BSH Electrodomésticos España S.A., Ctra. Castellón Km. 6.3, 50720 Zaragoza, Spain; roberto.latre@bshg.com

**Keywords:** gasket, transient state, accelerometer, load cell, washing machine, multibody

## Abstract

In horizontal-axis washing machines, the front gasket as well as the damping system are crucial owing to the possible collision of the tub with the housing during the transient period. However, most dynamic models for predicting tub motion focus on the steady state and consider only the suspension system without including the gasket. We conducted an experimental study to analyze the effect of the gasket on the transient motion of the tub. The results obtained indicate the necessity of implementing the gasket in the multibody model of a washing machine to accurately predict the tub behavior during this period. The gasket model is formed by a combination of Voigt elements. Stiffness parameters are determined using a load cell, and damping factors are estimated using a process that integrates Adams/View, Matlab optimization algorithms, and displacement measurements that are taken using accelerometers. A D-optimal design used to predict the effect of the gasket parameters reveals that the tub displacement is most sensitive to the changes in linear stiffness in the transversal direction. Finally, the model of the gasket provides a better approach for predicting the tub movement during resonance that can be used in the design phase to avoid tub collision.

## 1. Introduction

The design of a washing machine is challenging from the dynamic perspective because it is accentuated by the current trend of increasing the load capacity and spinning speed, as well as customers’ demand for noise comfort.

A horizontal-axis washing machine is typically formed by a tub that includes a drum that is driven by a motor, suspended with two or up to four springs that are attached to a housing. Furthermore, the tub is connected to the housing with two or up to five dampers at its lower portion and with a gasket at its front. The dynamic effect of the uneven distribution of clothes inside the drum is simplified to an eccentric load, which causes a tub motion of maximum amplitude during the startup ramp. In this stage, the drum speed passes through all rigid-body resonance frequencies of the system for an instant, which may cause it to become unstable.

During a washing machine cycle, three effects can arise: (i) the impact of the tub with the housing or other parts such as the detergent drawer; and as a result of the force transmission between the tub and the housing through the suspension system and the gasket; (ii) walking; and (iii) housing vibration, which result in noise emission.

Studies that investigate the motion control of an oscillating group by analyzing different balance and suspension systems [1] have been performed; additionally, design criteria to prevent housing walk [2] and reduce vibration [3,4,5] have been investigated.

First, simplified two-dimensional (2D) or three-dimensional mathematical models were established to understand these phenomena, primarily during the spin cycle. The numerical analysis of the developed set of equations resulted in simulation tools that were custom designed to analyze the effect of different design parameter values on washing machine performance and to predict the effects by the model. Computational advancements have allowed the use of several multibody dynamic software, such as Adams^®^ [6] or Modelica^®^ [7], for the modeling of washing machines. Using these tools, the modeling capabilities can be improved by introducing viscoelastic elements, e.g., the bushings between the damping system with the tub or the housing [8] that, owing to their complexity, are not considered in basic models.

As mentioned previously, current environmental issues have resulted in an increase in load capacity, which is concerning for the kinematics of the tub, as its motion space is increasingly reduced [9]. Therefore, many authors have focused on controlling the tub motion [10,11]. It is generally agreed that the function of the suspension system is to reduce the rigid body motion of the tub to avoid collision with the housing in the transient state [12]; hence, many models are focused on the design and understanding of the system. However, the door gasket, whose main role is to prevent water leakage, is overlooked in some models [13,14], even though it introduces forces on both the tub, which can modify its motion [15], and the housing.

Regarding the dynamic models of horizontal-axis washing machines found in the literature, which introduce both the suspension system and the gasket, the model presented by Türkay et al. [16] predicts, for a new suspension design, the amplitudes of the tub during transient and steady states as well as the liftoff and sliding of the housing, within acceptable errors. The gasket is included as two orthogonal springs in a plane parallel to the door expressed by nonlinear functions and obtained from experimental data without considering the forces and torque in other directions. In this study, the possible sources of discrepancies between the experimental and simulation results are discussed, but the effect of the gasket is not analyzed. Baykal et al. [17] modeled a door gasket with two orthogonal springs and a spring perpendicular to the front of a cabinet. To test the effectiveness of the model, two cases involving different values of gasket parameters were studied without specifying the method in which those values were determined.

Wagner and Pfeiffer [18] developed a mechanical model that considers both rigid body motion and elastic vibrations. The authors proposed six spring–damper elements for the door gasket. Considering a linear behavior, the coefficients of the stiffness matrix were determined via static finite-element analysis; however, neither the method to adjust the damping coefficients nor the gasket behavior were detailed in their paper.

Yumak [19] studied the dynamics of an inclined oscillating group modeled with Adams^®^. The author reported that the door gasket served as a nonlinear spring and damping element, in which damping was disregarded as it was assumed to be relevant only at high speeds. The proposed model was formed using eight spring elements with six degrees of freedom between the tub and housing with a 45${}^{{}^{\circ}}$ angle. Static tests to determine the stiffness were not presented, neither was the effect of the gasket on the system dynamics.

Argentini et al. [15,20] developed a parametric multibody model of an oscillating group with dry friction dampers at the steady state. The parameters of these dampers were modified to investigate new designs for the suspension system. The authors reported that the door gasket was difficult to model because of its nonlinear behavior; however, in their study, they simplified the model as a linear elastic and dissipative element placed at the center of the porthole. The unknown parameters, such as the stiffness and damping of the suspension system and the gasket, were obtained by minimizing the error between the numerical and experimental dissipated energies. However, the authors did not provide any other information regarding the gasket.

Drüke et al. [21] modeled the door gasket as a linear spring–damper element as well. They focused on the potential causes of rotordynamic instabilities without evaluating the effect of the gasket.

Gündüz [22] did not include the gasket in the mathematical modeling of a horizontal axis washing machine because, despite its damping and spring characteristics, the author disregarded the effect of the gasket on the movement, unlike the main oscillation of the system.

Nygards and Berbyuk [23] built a multibody model using Adams^®^ and explained the tests applied to identify the parameters in detail; however, they assumed that the door was fixed to the tub and merged a door-seal system with the tub into one body. Buśkiewicz et al. [24] used this approach as well, in which the mass of the gasket was included in the tub unit, but its flexibility was disregarded. For future work, the authors proposed introducing the elastic and dissipative characteristics of the joints and door gasket to improve the correlation between numerical and experimental data.

Boyraz and Gündüz [25] developed a 2D model and optimized both the transient and steady-state vibration characteristics using genetic algorithms, without considering the stiffness effects of the door gasket.

In designing a washing machine, the forces transmitted to the housing must be reduced; therefore, most previous studies analyzed the housing vibration and discussed the mathematical modeling methods of the suspension system; consequently, new solutions were proposed. Although some authors are aware of the effect of the door gasket for mitigating the varying gap between the tub and housing as well as for absorbing vibrations, this aspect is less emphasized. Furthermore, it is challenging to relate the characteristics and dynamic performance of the gasket. Hence, because the interaction between the gasket and tub is inevitable, additional studies are necessitated to further understand the door gasket behavior.

The aim of this study is to predict the tub motion of a front load washing machine more accurately during the transient to ensure the gap calculation between the tub and the housing owing to space limitations, on average between 20 mm and 40 mm. The research methodology followed in the study begins with an extension of the initial multibody model of an oscillating group that included a suspension system, built in Adams^®^ by the authors, with the mathematical model of the door gasket identified from experimental characterization. The new model allows us to determine the sensitivity of the gasket dynamic behavior based on its design parameters by using a D-optimal design to screen their effects.

This paper is organized as follows: in Section 2, we present the effect of the gasket on the motion of the oscillating group, as determined experimentally. Section 3 describes the method to develop a kinetic model of the gasket to be included in the model of the washing machine and used for model validation. Finally, the sensitivity analysis of the model and the conclusions are presented in Section 4 and Section 5, respectively.

## 2. Experimental Characterization of Gasket Performance

In this section, we present the experiment conducted to analyze whether the door gasket must be considered in the multibody model of a horizontal-axis washing machine. The first aim of this experimental analysis was to determine the effect of the gasket on the dynamic behavior of the tub. The second aim was to obtain the displacement data such that the parameters of the oscillating group model can be adjusted and validated [26].

### 2.1. Measurement Setup

**Figure 1:**
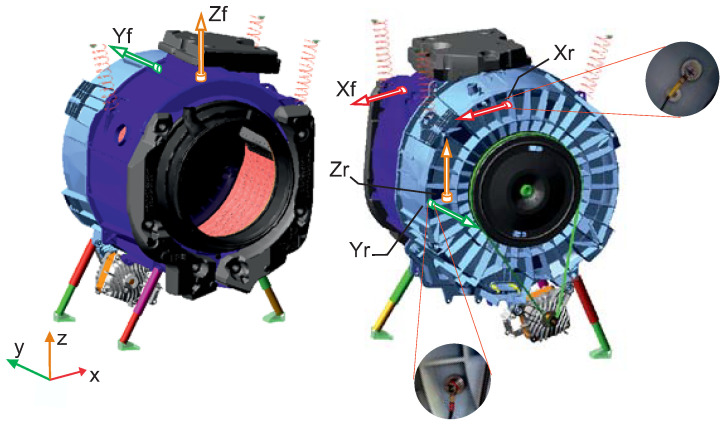
Measurement points on the oscillating group.

The front load washing machine selected for the experimental setup is shown in Figure 1. It comprises a plastic tub suspended from the housing by three upper springs and three dry friction dampers at the bottom. One upper and two front counterweights stabilize its movement, and a ring-shaped gasket attaches the tub along its perimeter to the front panel of the housing to prevent water leakage. Inside the tub, a drum is supported by two bearings at the end of the shaft. A brushless direct current motor drives the drum shaft through a two-pulley system.

**Figure 2:**
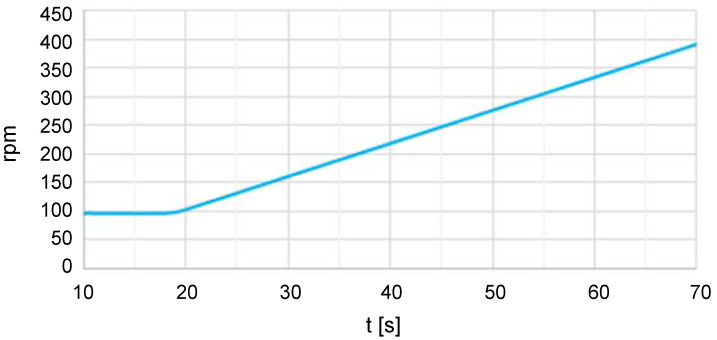
Speed ramp.

The oscillating group was tested with and without the door gasket to assess the effects. As the laundry distributes unevenly within the drum, we analyzed two loading conditions with magnets located at different positions in the drum, i.e., a middle load of 1 kg and a counter load of 2 × 0.5 kg to consider the static and dynamic unbalance, respectively. According to the manufacturer’s test protocol, we measured the displacement of the tub at six points, i.e., three at the rear of the tub, ($X_{r}$, $Y_{r}$, $Z_{r}$), and three at the front part, ($X_{f}$, $Y_{f}$, $Z_{f}$). Figure 1 shows the positions and measurement directions. The displacements of these points were recorded using piezoelectric accelerometers connected to multianalyzer Pulse 3160-A-042, which was controlled by a computer with PULSE^®^ LabShop software from Hottinger Brüel & Kjær company. The tests were performed for 70 s, and the speed of the drum was increased from zero to 400 rpm using a low constant acceleration of 7 rpm/s, as this setup enabled the resonance step to be prolonged and the maximum displacements to be analyzed more effectively. Figure 2 presents the velocity ramp where the frequency corresponding to each time can be determined. Each measurement was repeated five times.

### 2.2. Results

**Figure 3:**
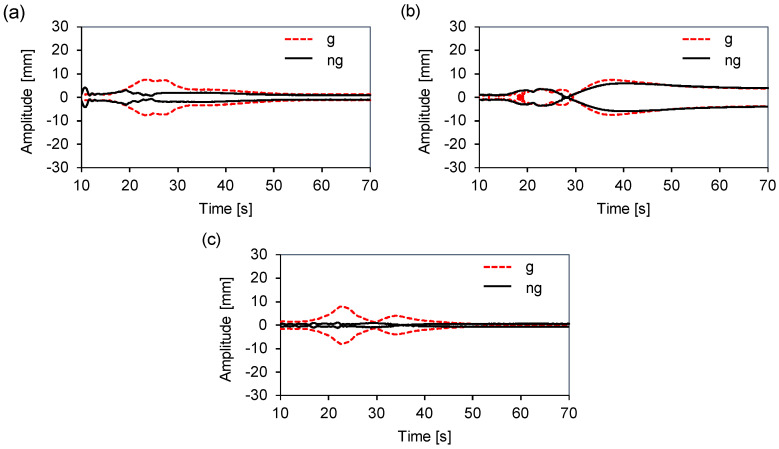
Displacement envelopes: (**a**) $X_{f}$ counter load, (**b**) $Z_{r}$ counter load, (**c**) $Y_{f}$ middle load.

To facilitate the comparison of the recorded signals, after applying 0.5 and 20 Hz fourth-order Butterworth filters, we developed an application in Matlab^®^ to obtain the upper and lower envelopes of the displacement signals in the time domain. In Figure 3, three out of the twelve displacement envelopes obtained are displayed. To detect the points at which the greatest differences between the envelopes may occur for each loading condition, which will be analyzed in more detail in the future sections, we propose to use the maximum amplitude as an indicator.

Subsequently, for each point $i\in \{X_{r},\;Y_{r},\;Z_{r},\;X_{f},\;Y_{f},\;Z_{f}\}$ and under each scenario j∈{g(withgasket),ng(withoutgasket)}, the maximum amplitude, $A_{i}^{j}$, of the tub was calculated. To evaluate the effect of the gasket on the dynamic behavior of the tub, the relative difference in percentage $\Delta_{i}$ between $A_{i}^{g}$ and $A_{i}^{ng}$ was computed at each point:

(1) $$\Delta_{i}\left(\%\right)=\frac{A_{i}^{g}-A_{i}^{ng}}{A_{i}^{ng}}\times 100$$

Table 1 shows the percent difference for both loads at each point for the six points. It is clear that the gasket affected the tub movement and, as expected, the effect differed depending on the loading condition. The maximum displacement amplitudes occurred primarily in the *x*–*z* plane in the case of the middle load, whereas the counter load tilted up the tub and produced a gyroscopic effect. The moment arising from this tilt yielded a precession that turned the tub in the *x*–*y* plane.

As shown by the measurement results, for the middle load, the gasket increased the displacement of all points, except for point $X_{f}$. Greater differences were obtained for points $Y_{r}$ and $Y_{f}$ particularly at the front since the displacements of these points were negligible if no gasket were used.

The effect of the gasket was less significant in the case of the counter load. It is noteworthy that the displacements of the tub under a counter-loading condition were smaller than those for the middle load in the *x*- and *z*-directions. On the contrary, the displacement increased in the *y*-direction at the front, with and without the gasket, and at the rear without the gasket. For the counter load, the most significant difference occurred at the front in the *x*-direction. The effect of the gasket was negative at points $X_{r}$ and $Y_{f}$, i.e., the amplitude decreased.

Higher displacement differences on the three axes, with and without the gasket, were indicated at point $Y_{f}$ for the middle load and at points $X_{f}$ and $Z_{r}$ for the counter load. Figure 3 shows a comparison of the envelopes at these points, where the rigid-body resonance modes were excited during the startup ramp in a speed ranging from 100 to 300 rpm. At speeds lower than 300 rpm, the displacement increased up to 80% on the *x*-axis, and up to 605% on the *y*-axis, whereas on the *z*-axis, the effect was less significant, with a maximum increase of 25%. Based on this speed, we observed that the displacement level was steady; therefore, from a dynamical viewpoint, the gasket is negligible during the steady state as it does not interfere with the natural movement of the oscillating group during the spinning cycle. The conclusion is that the gasket operates primarily during the resonance period when the largest displacements and rotations occur. In this step, the contact between the different folds increases its stiffness as well as the forces that are exerted on the tub which modify the dynamical behavior of the oscillating group.

Previous studies regarding washing machine dynamics are primarily focused on spinning. Hence, many authors disregard the effect of the door gasket and do not include it in the washing machine model because the gasket function is to seal the tub. However, we discovered that this assumption is only valid for the steady state. On the contrary, the gasket increases the displacement of the tub during the transient period. Owing to the effect of the gasket on the tub motion during the transient state, it is important to develop a kinetic model to achieve a more precise prediction of the dynamic behavior of the oscillating group.

## 3. Multibody Model of the Oscillating Group

The main challenge in the development of the dynamic model of a washing machine is the characterization of nonlinear elements, such as the suspension system and gasket. Because of the complexity of modeling both components in parallel, we first defined a multibody model without the gasket to adjust the parameters of the suspension system; subsequently, the gasket model was implemented. As the focus of this study is to develop a model for the gasket, we herein briefly describe this initial dynamic model of the front load washing machine without detailing the characterization of the parameters of the suspension system. We used the multibody software Adams^®^ from MSC Software Corporation to model the oscillating group dynamics.

One degree of freedom, $\phi_{D}$, defines the spinning of the drum, and six degrees of freedom, expressed by the coordinate vector $q=[X_{G},\;Y_{G},\;Z_{G},\;\psi ,\;\theta ,\;\phi ]$ models the center of mass and the 3-2-1 Euler angles of the tub. The group was modeled as two rigid bodies connected by a revolute joint; a solid, driven by the motor, was formed by the drum with the shaft, inner track, bearing balls, upper pulley, and unbalanced load. The second body included the tub, hub, and outer track of the bearings, counterweights, and motor.

The oscillating group, as shown in Figure 1, incorporates three upper springs attached at their ends to the tub and the housing through pin joints, and three dry-friction dampers at the bottom. Hooke joints were used to model the viscoelastic bushings that connect the dampers to the tub and housing. For the damper friction force, we defined a nonlinear model that considers the properties of the friction sponge [27], and the bushings were modeled with spring-damper elements and a friction torque to consider sliding. We defined the housing as a ground part, which is suitable for the analysis, and assumed that the motor provided the defined angular velocity independently of the necessary torque. To create the model, the mass and inertia properties required by the equations of motion were calculated using the part geometry and density.

**Figure 4:**
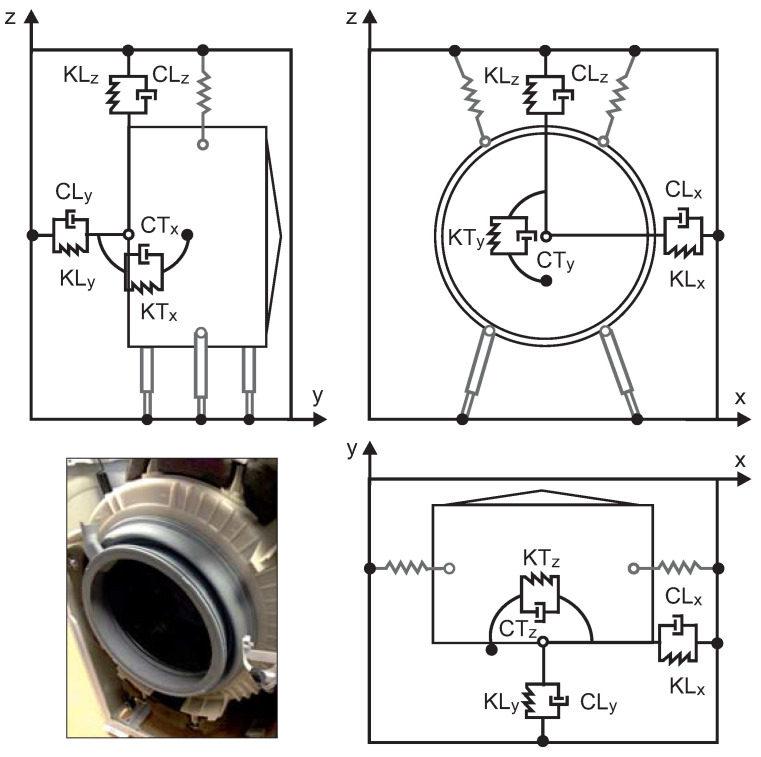
Gasket model.

Subsequently, we extended this initial multibody model with the gasket to improve the prediction of the movement during the transient state. Gaskets are made of rubber, which is a viscoelastic material. Mechanical models are often used to model the viscoelastic behavior of materials of varied biological [28] and engineering applications [29]. The Kelvin–Voigt model is one such model that combines spring and viscous damper in parallel to model the elastic behavior and energy dissipation, respectively. In this assembly, the spring and damper expand simultaneously and demonstrate the primary creep phenomenon. Under force application, the deflection is not instantaneous; this is because of the damper. The deformation progressively grows while the spring gradually shares the load. When unloaded, the spring will not recover its initial position immediately and will apply a force on the damper; hence, a compressive creep will occur [30]. Eventually, the creep strain will be recovered after a finite time. For the right operation of the gasket, the retention of the sealing force is crucial. Therefore, the nonlinear material properties of the rubber gasket were described using an empirical model that was defined as a massless interface between the tub and the housing. The gasket model was defined using a combination of springs and dampers to represent the rubber behavior. The gasket must exert a force torsor that does not prevent any of the six degrees of freedom of the tub. After testing several options, we decided to use the model shown in Figure 4.

For each direction d∈{x,y,z}, we defined a linear ($KL_{d}$, $CL_{d}$) and a torsional ($KT_{d}$, $CT_{d}$) Voigt model attached to the front of the tub; this enabled tub translation and rotation at the three axes. The next section describes the method by which the parameters of this gasket model were determined.

### 3.1. Testing of Gasket Model Parameters

Because the model of the door gasket was integrated in a multibody dynamic software, the gasket was characterized at the component level rather than at the material level. Many factors affect the gasket behavior, i.e., not only the material properties, but also the geometry and initial interference. Hence, we tested the gasket as it was placed in the washing machine to determine the parameters required for the multibody simulation. Quasi-static tests enabled us to estimate the stiffness characteristics of the gasket model. Subsequently, we compared the displacement measurements of the tub with the gasket, as presented in the experimental study of Section 2, with the multibody predictions to determine the unknown damping parameters.

#### 3.1.1. Stiffness Parameters

**Figure 5:**
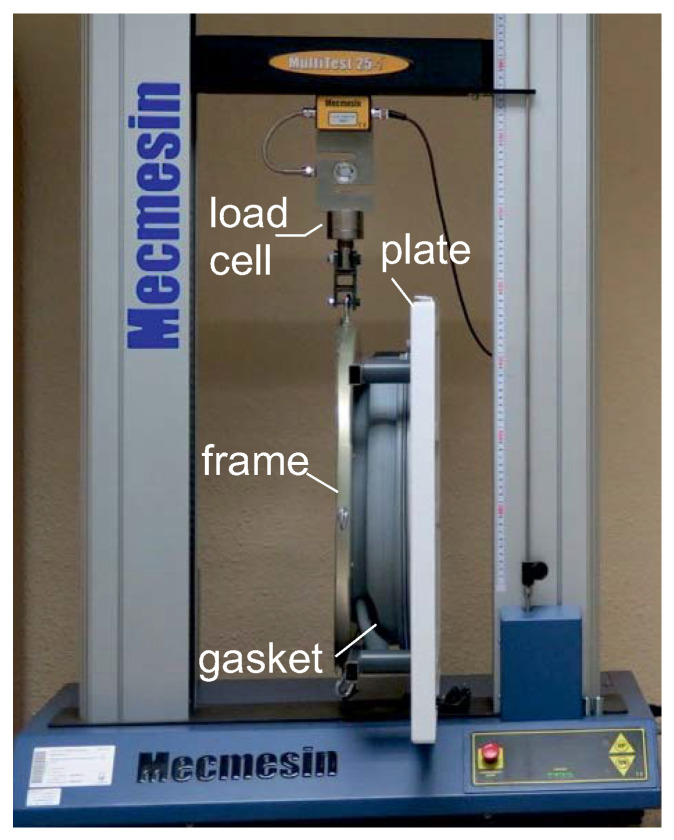
Test setup.

Using a test rig designed to rotate and translate the gasket such that the torsional, axial, and radial movements are uncoupled, we performed various experiments to determine the elastic parameters of this element. To simulate the washing machine procedure, the gasket was clamped at the rest position between a base plate and part of the tub front that was fixed to a metal frame (see Figure 5).

A 25 kN load cell sensor of a tensile testing machine (Mecmesin, Multitest-i 25 model) was attached to this metal frame at different positions. Depending on the test performed, the testing machine shifted this metal frame such that the gasket underwent axial, radial, or torsion deformations.

First, to characterize the torsional stiffness parameters, $KT_{d}$, the gasket was rotated about the corresponding *d*-axis, d∈{x,y,z}. Next, we applied in-plane forces at the two orthogonal directions, *x* and *z*, and a force at the *y*-axis perpendicular to the base plate to identify the radial $KL_{x}$, and $KL_{z}$ as well as axial $KL_{y}$, stiffness factors, respectively. The tests were conducted at room temperature with a constant velocity of 100 mm/min, and were repeated thrice.

**Figure 6:**
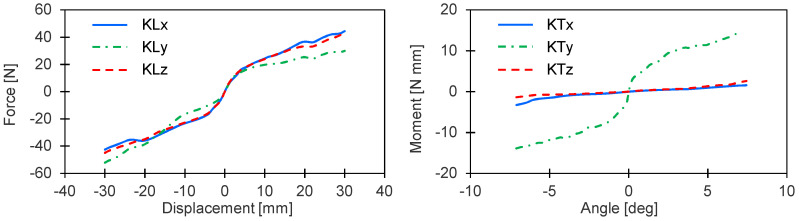
Stiffness splines.

The experimental results are summarized in Figure 6, which shows the force vs. the displacement and the torque vs. the angle. The nonlinear gasket responses in compression and tension were equivalent; furthermore, we observed similar behaviors in the *x*- and *z*-directions owing to the symmetry of the ring shaped gasket. On the *y*-axis, the gasket was stiffer under rotation but more flexible under linear displacement. These measured stiffness splines were implemented in the multibody model before the damping parameters were determined in the next step.

#### 3.1.2. Estimation of Damping Parameters

An appropriate estimation of damping parameters is essential to accurately describe the dynamics of the system. To simplify the model, we assumed that the linear and torsional parameters were of the same value in all directions; therefore, only two values, $C_{L}$ and $C_{T}$, must be estimated. This assumption greatly reduces the computational effort because the optimization problem is on two parameters instead of six parameters. As shown in Section 3.2, the fitted model allows us to predict the motion of the tub with sufficient precision. To simplify the notation, we denote vector $\left(C_{L},\;C_{T}\right)$ of the damping parameters by Θ.

The aim is to obtain the value of Θ to achieve the maximum similarity between the simulated and experimental displacement envelopes. Hence, the difference between the numerical and experimental results presented in Section 2 was considered in Equation (2), for both loading conditions, k∈{middle,counter}. We considered the tub displacement at each of the six points $X_{r},\;Y_{r},\;Z_{r},\;X_{f},\;Y_{f}$ and $Z_{f}$, for both loads when the tub displacements were higher, i.e., from 10 to 50 s, with a time step of Δt=0.005 s as follows:

(2) $$f_{k}\left(\Theta \right)=\sum_{i=1}^{6}\sum_{t=10}^{50}{\left(A_{kit}^{mb}-A_{kit}^{exp}\right)}^{2}$$

where $A_{kit}^{mb}$ and $A_{kit}^{exp}$ denote the displacement values obtained by the multibody model and experimental tests, respectively, at point *i* and time *t*, for the *k*-loading condition. The function $f_{k}$ expresses the distance in terms of the 2-norm. We aim to minimize the distance between the experimental data and the simulated results for both loading conditions. Hence, we addressed this bi-objective optimization problem using a weighted-sum method, in which both objective functions are equally weighted as follows:

(3) $$f\left(\Theta \right)=f_{\mathrm{middle}}+f_{\mathrm{counter}}$$

**Figure 7:**
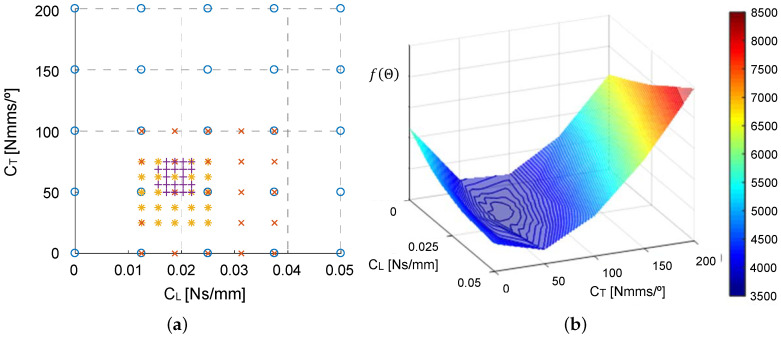
Grid optimization method: (**a**) grid of discrete points on Ω; (**b**) surface plot of function f(Θ).

Several optimization methods [31], such as the bisection method or the gradient method for optimization, can be used to minimize *f*. However, we considered a grid optimization method, which is a derivative-free method. This method provides a sufficiently favorable solution in a short computational time. Based on the experimental tests, we restricted our attention to the interval [0,0.05] Ns/mm for $C_{L}$ and [0,200] Nmms/${}^{{}^{\circ}}$ for $C_{T}$. These intervals provided the region of interest, denoted by Ω, for vector Θ. An Adams^®^ -Matlab^®^ co-simulation process allows us to approximate the objective function *f* on the domain Ω and to focus on the promising areas of Ω. The optimization process begins with this domain discretized into a uniform grid with 25 points, as displayed as circles in Figure 7a. A grid search iteration involved considering for every grid point $\left(C_{L}^{m},\;C_{T}^{m}\right)$, the model developed in Adams^®^. This model was executed using the damping parameter values provided by the grid point to obtain the displacement values and to compute the corresponding value of the objective function f. We selected the grid point with the best objective function value for the next step. A half-size refined mesh was defined around the selected grid point (cross marks in Figure 7a), and a new grid search iteration is implemented. The successive iterations refined the mesh, as shown in Figure 7a. The process was terminated when there was no progress on the minimization of f(Θ); this happens when the difference between the value of the objective function *f* computed in two consecutive iterations becomes lower than 0.02. A surface plot of the objective function *f* is displayed in Figure 7b. For the values of the damping parameter $C_{L}$ = 0.019 Ns/mm and $C_{T}$ = 60 Nmms/${}^{{}^{\circ}}$, the function *f* yielded the lowest value; therefore, it was selected as the optimal solution.

The results yielded by the multibody model, which considers the optimal damping parameters above, agreed well with the displacement results measured experimentally under both unbalanced loads. By analogy, we used Equation (1) to compute the percent difference $\Delta_{i}$ between the maximum amplitudes provided by both scenarios j∈{mb(multibodymodel),exp(experimentaldata)}, for each point $i\in \{X_{r},\;Y_{r},\;Z_{r},\;X_{f},\;Y_{f},\;Z_{f}\}$.

**Figure 8:**
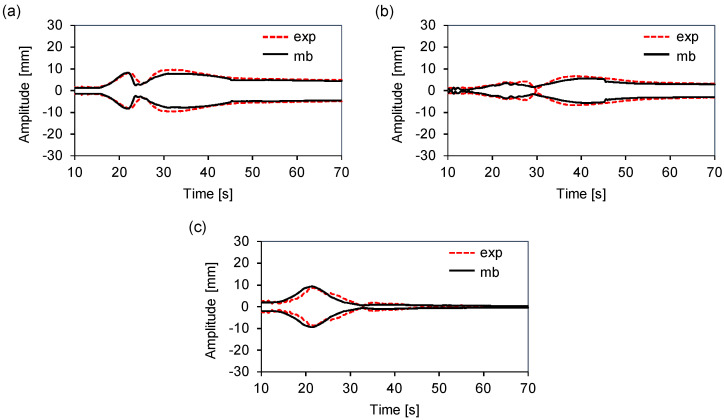
Multibody and experimental displacement envelopes: (**a**) $X_{r}$ counter load. (**b**) $Z_{f}$ counter load. (**c**) $Y_{r}$ middle load.

Table 2 displays those values. For the middle load, the differences were between −10.1% at $Z_{f}$, and 7.4% at $Y_{r}$. In the case of the counter load, the multibody model underestimated the displacement amplitude at all times, with a difference ranging from −15.3% at $Z_{f}$ to −1.0% at $Y_{f}$. Figure 8 shows a comparison of the displacement envelopes for the middle load at $Y_{r}$, and for the counter loads at $X_{r}$ and $Z_{f}$. The multibody model captured the tub movement during the transient. Although the resonance modes shifted slightly, both signals were similar. The resonance frequency is governed by the stiffness of the system. Hence, the sources of mismatch can be associated with multiple physical phenomena not directly included in the mathematical model, such as the method used to determine the stiffness of the springs. The tests were performed such that the movement was independent in each of the three directions. However, it was difficult to completely uncouple the rotation or translation along each axis. Figure 8 shows the excellent agreement between the tub displacement measurements and the dynamic multibody results during the steady state.

### 3.2. Model Validation

The damping parameters in the model were estimated based on experimental tests performed with middle and counter loads. However, because the laundry is randomly spread within the drum, industrial protocols’ test loads placed in the front or at the back of the drum as well because they combine both dynamic and static unbalance. However, whether our multibody model can accurately predict the movement under these loading conditions is yet to be elucidated. To validate the multibody model, we computed the displacement envelopes with two new loading conditions for the six tub points, i.e., a load of 0.7 kg placed at the back and at the front of the drum. Furthermore, we obtained the displacement envelopes based on the experimental procedure explained in Section 2.

Table 3 displays the peak amplitude values and the percent difference between the multibody model results and the experimental data. For both loads, the maximum displacement of the tub was in the *x*–*z* plane, except for the front load at the rear of the tub where the highest displacement was in the *x*–*y* plane. The comparison between the maximum values of the numerical and measured envelopes, indicates that the difference was lower than 17% for the back load and 13.1% for the front load; additionally, a close agreement between 2 and 3% was observed at some points.

**Figure 9:**
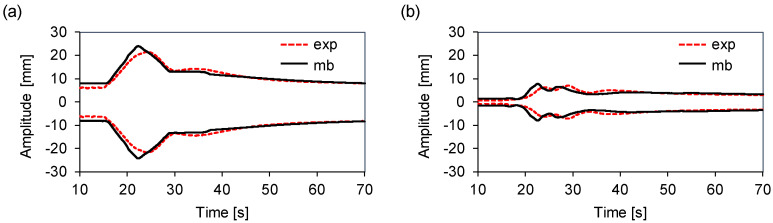
Multibody and experimental displacement envelopes: (**a**) $X_{r}$ back load; (**b**) $Y_{f}$ front load.

Figure 9 displays the multibody and measured envelopes at points $X_{r}$ and $Y_{f}$ for the back and front loads, respectively. In both the transient and steady states, the predicted values agreed well with the experimental data. These results confirm that our assumption on the damping coefficients simplifies the computational effort required to fit the model while providing an accurate prediction of the motion of the tub.

Hence, the door gasket should be implemented in the multibody model of an oscillating group as this enables washing machine designers to gain further insights into the mechanism during the transient state.

## 4. Sensitivity Analysis

The main concern is the analysis of the collision of the tub and the forces transmitted to the housing during the transient state. With the door gasket incorporated into the multibody model of the oscillating group, the aim of the sensitivity analysis is to determine the effect of the damping and the stiffness parameters of the gasket on the movement of the tub point nearest to the detergent box, as well as the forces transmitted to the housing by the gasket, for the middle and counter loads.

The door gasket model included linear $C_{L}$ and torsional $C_{T}$ damping parameters and six stiffness parameters, three linear at each direction, $KL_{x}$, $KL_{y}$ and $KL_{z}$; and three torsional, $KT_{x}$, $KT_{y}$ and $KT_{z}$. These parameters were considered as factors of a design of experiments (DOE). This statistical methodology aims to decrease the number of experiments required to identify variables affecting the response variable values. To achieve the aim of this analysis, we introduced the following response variables in each direction d∈{x,y,z}:

- $D_{d}$ are the maximum displacements [mm] of the tub point closest to the detergent box.
- $F_{d}$, and $M_{d}$ are the maximum forces [N] and moments [Nmm] transmitted to the housing by the gasket.

**Figure 10:**
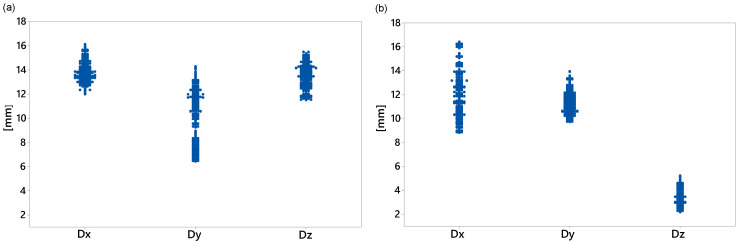
Maximum displacements of point closest to detergent box: (**a**) middle load; (**b**) counter load.

**Figure 11:**
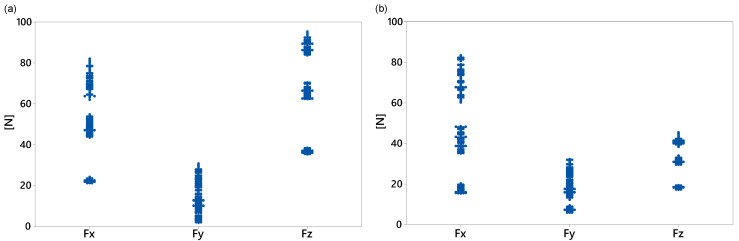
Maximum forces transmitted to housing by gasket. (**a**) middle load; (**b**) counter load.

**Figure 12:**
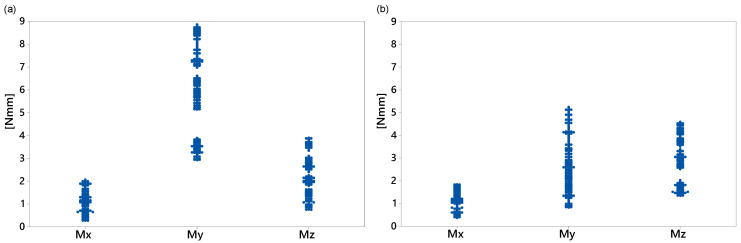
Maximum moments transmitted to housing by gasket: (**a**) middle load; (**b**) counter load.

The DOE provides the most effective combination of the factors. In this study, our focus is on three levels for each parameter, i.e., −1 (low), 0 (nominal), and 1 (high). The damping nominal values were the optimal values obtained in Section 3.1.2. To reduce the computational time, linear and torsional stiffness nominal values were calculated as slopes of linear fit to the first 20% of the force–displacement and torque–angle curves, respectively. All factors were bounded by 50% of their corresponding nominal values. The full DOE of this study comprised $3^{8}=6561$ experimental trials for each loading condition. However, we used the D-optimal design generated by Matlab^®^ with 1000 trials for each loading condition to simultaneously screen the three factors. For each factor combination, the multibody model developed in Section 3 was executed, and all nine response variables were computed. As expected, the dynamic performance of the washing machine depended on the load type, in particular for the displacement response variables. Figure 10, Figure 11 and Figure 12 show all the data collected for the combinations of factors provided by the D-optimal design. The *x*-axis data indicate the name of the response variable whose values are displayed on the vertical axis.

Figure 10 shows the maximum displacement during the transient state at each direction for both loading conditions. For the middle load (Figure 10a), the lowest displacement, but with more variability, was on the *y*-axis. The maximum displacement data in the *x*- and *z*-directions were similar and ranged from 11.5 to 16.1 mm. However, for the counter load (Figure 10b), the smallest maximum displacement with the least variability in the data was in the *z*-direction and ranged from 2.1 to 5.2 mm. Figure 11 and Figure 12 show some data clusters. An analysis of variance (ANOVA) can identify the factors that significantly affect the response variables and the level of those factors that yield the best values for the response variables.

In this study, we considered the main effects and second-order interactions. Table 4 shows the percent contribution of the main effects and second-order interactions to explain the variance of the response variables. For all the response variables and both loading conditions, except for $D_{x}$ under a middle-loading condition, the main effects contributed to at least 92.5% of the output responses. Although several second-order interactions are statistically significant, their contribution for explaining the output response variability was small compared with the main effects.

Table 5 displays the most relevant information obtained from the ANOVA. Only the main effects and the second-order interactions with a contribution greater than 10% and 5%, respectively, are shown in the table for each response variable. The first column shows the name of the response variable. The second to fifth columns display information pertaining to the middle-loading condition, where the constant term of the adjusted model, $\beta_{0}$, represents the average of the response variable when the significant factors are on level 1. The third column includes the significant factors and their corresponding percent contributions, in parentheses, to explain the output response variability. The fourth and fifth columns show the effects of −1 and 0 levels of the factor on the mean value $\beta_{0}$.

**Figure 13:**
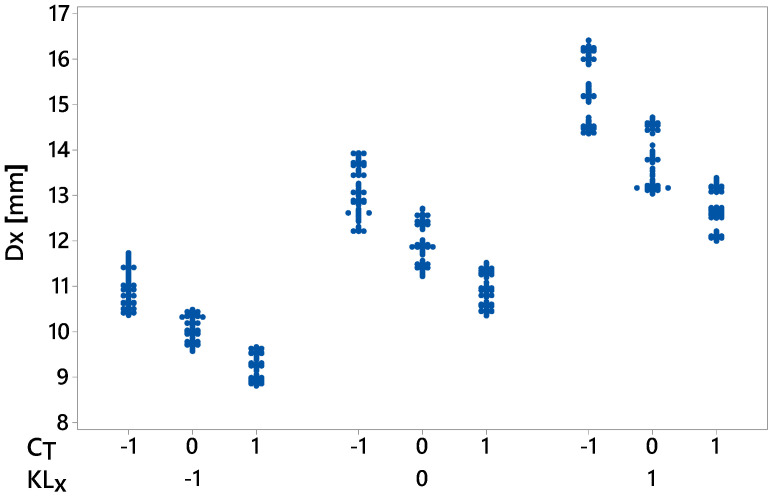
Maximum displacement in the *x*-direction with counter load.

For instance, the mean value of $D_{x}$ with counter-loading condition was 11.99 mm. The most influential factor was $KL_{x}$, which contributed to 70.64% of $D_{x}$ variability. If factor level −1 is selected, then the mean value would be reduced by 1.91 mm. These effects are displayed in Figure 13. In this chart, the values of the response variable $D_{x}$ collected by the D-optimal design are displayed in the vertical axis for each factor level combination of $KL_{x}$ and $C_{T}$ shown on the *x*-axis. Decreasing the value of $KL_{x}$ and increasing the value of $C_{T}$ can reduce the maximum displacement in the *x*-direction more effectively.

Summarizing the information provided in Table 5, we conclude the following: torsional stiffness $KT_{x}$ and $KT_{z}$ were insignificant factors for any response variable. Therefore, the gasket model can be simplified for future analyses. However, the torsional stiffness $KT_{y}$ only affected the response $M_{y}$ for both loading conditions. Decreasing the value of $KT_{y}$ resulted in a decrease in the maximum moment in the *y*-direction. In the cases of response variables $M_{x}$ and $M_{z}$, torsional damping $C_{T}$ was the most influential factor. With a middle load, $KL_{x}$ affected $M_{z}$ as well. In all cases, the level −1 of $C_{T}$ and $KL_{x}$ reduced the maximum moments.

For both loading conditions, the force analysis revealed that the most influential gasket factor in each direction was the linear stiffness in the corresponding axis, and that the lowest bounds provided the minimum force values. For instance, in the middle-loading condition, the mean value of 63.48 N of $F_{z}$ decreased by 26.83 N with a level −1 of the $KL_{z}$ factor.

The analysis of the maximum displacement during the transient indicated that the most significant model parameters were the linear stiffness $KL_{x}$ followed by the torsional damping $C_{T}$. In general, low values of $KL_{x}$ and high values of $C_{T}$ tend to attenuate the displacement amplitude. However, low $C_{T}$ values decrease the torque, contrary to the fact that increasing the damping decreases the displacement amplitude during resonance, resulting in a trade-off between torque and displacement attenuation.

Moreover, $C_{L}$ is an influential factor on $D_{x}$ for the middle load and on $D_{y}$ and $D_{z}$ for counter load. In all cases, the smallest displacement occurred with high values of the factor. The linear stiffness $KL_{y}$ affected $D_{y}$ for the counter load, and the lowest bound reduced the displacement easily. However, reducing $D_{z}$ for both loading conditions implies a high level of the influential factor $KL_{z}$. This conflicts with the reduction of forces in the *z*-direction.

## 5. Conclusions

The growing demand for washing machines with higher capacity load is an extremely challenging issue for appliance design engineers, as the reduction in gap between the tub and housing increases the possibility of collision between both, mainly in the transient state. The current trend is to use multibody models to predict the dynamic behavior of this appliance. However, most of these models consider only the suspension system and disregard the gasket, which is fundamental during the transient period, as demonstrated in our experiment.

The main contribution of this study is the development of a kinetic model of the door gasket and the characterization of its viscoelastic parameters based on experimental data obtained using accelerometers and a load cell sensor. Knowledge of the gasket performance is essential to the design process and enhances the approximation to the real oscillating group motion during resonance compared with conventional prediction models. The results of this study may facilitate washing machine manufacturers in reducing tub movement while increasing load capacity.

The sensitivity analysis revealed that motion of the tub and the force and moment transmission to the ground are more sensitive to linear stiffness parameters than torsional stiffness parameters, with the most relevant parameter being the linear stiffness in the transversal direction. Low levels of these factors reduced the tub displacement, forces and moments. Torsional damping was another relevant factor but had the opposite effect on tub displacement and moments, i.e., high levels of torsional damping reduced the displacement but increased moment transmission resulting in a trade-off between torque and displacement attenuation. The tub displacement was also reduced for high levels of linear damping parameter.

The gasket was characterized based on a combination of multibody simulations and experimental tests; therefore, testing any new gasket design requires the building of prototypes to identify the appropriate parameters for the mathematical model. Hence, the development of a procedure to relate the geometrical and material properties of the gasket to the stiffness and damping parameters of its mathematical model offers a new research avenue that may be advantageous for comparing multiple virtual designs and enabling a more optimized usage of prototypes.

## Figures and Tables

**Table 1:** Experimental amplitudes and percent difference with/without gasket.

	Middle Load						Counter Load					
Variable	$X_{r}$	$Y_{r}$	$Z_{r}$	$X_{f}$	$Y_{f}$	$Z_{f}$	$X_{r}$	$Y_{r}$	$Z_{r}$	$X_{f}$	$Y_{f}$	$Z_{f}$
$A_{i}^{ng}$ [mm]	23.6	3.2	18.3	24.2	1.1	20.0	12.6	12.8	6.0	4.2	13.5	3.5
$A_{i}^{g}$ [mm]	29.1	8.7	18.9	23.9	8.0	23.0	9.6	12.9	7.4	7.6	11.3	4.3
$\Delta_{i}$ [%]	23.2	175.3	3.3	−1.0	604.5	14.9	−23.7	1.0	25.0	80.1	−16.3	23.9

**Table 2:** Middle and counter loads: Percent displacement difference multibody/experimental.

Case	$X_{r}$	$Y_{r}$	$Z_{r}$	$X_{f}$	$Y_{f}$	$Z_{f}$
Middle load	4.4	7.4	−3.3	−1.9	7.0	−10.1
Counter load	−14.4	−3.5	−13.1	−3.6	−1.0	−15.3

**Table 3:** Back and front loads: Amplitude and percent difference of multibody/experimental results.

	Back Load						Front Load					
Variable	$X_{r}$	$Y_{r}$	$Z_{r}$	$X_{f}$	$Y_{f}$	$Z_{f}$	$X_{r}$	$Y_{r}$	$Z_{r}$	$X_{f}$	$Y_{f}$	$Z_{f}$
$A_{i}^{exp}$ [mm]	21.5	8.7	11.6	10.3	8.2	9.3	12.9	11.3	7.1	11.7	7.1	13.1
$A_{i}^{mb}$ [mm]	24.1	8.1	11.9	12.1	8.7	9.1	11.6	12.6	7.7	10.2	7.9	13.3
$\Delta_{i}$ [%]	11.9	−7.3	2.8	17.1	5.9	−2.5	−10.4	11.2	8.4	−13.1	10.9	1.8

**Table 4:** Percent contribution of main effects and second-order interactions.

	Middle-Loading Condition		Counter-Loading Condition	
	Main Effects	Interactions	Main Effects	Interactions
$D_{x}$	68.42	29.41	98.74	1.19
$D_{y}$	98.97	0.93	95.77	3.46
$D_{z}$	92.50	7.13	92.98	6.04
$F_{x}$	99.23	0.72	99.14	0.84
$F_{y}$	94.29	5.63	98.36	1.49
$F_{z}$	99.77	0.21	99.69	0.23
$M_{x}$	95.48	4.41	96.92	2.72
$M_{y}$	99.37	0.60	95.28	4.43
$M_{z}$	95.09	4.89	99.59	0.40

**Table 5:** Terms, percent contribution, and coefficients for each response variable and loading condition.

	Middle-Loading Condition				Counter-Loading Condition			
Response	$\beta_{0}$	Factor	−1	0	$\beta_{0}$	Factor	−1	0
$D_{x}$	13.72	$C_{L}$(27.92%)	0.48	0	11.99			
		$C_{T}$(17.42%)	0.40	−0.07		$C_{T}$(22.65%)	1.13	−0.07
		$KL_{x}$(13.96%)	−0.30	0.35		$KL_{x}$(70.64%)	−1.91	0
		$C_{T}*KL_{x}$(8.98%)						
		$KL_{x}*KL_{z}$(9.92%)						
$D_{y}$	10.23				11.30	$C_{L}$(14.37%)	0.40	−0.04
						$C_{T}$(63.55%)	0.82	−0.05
		$KL_{x}$(88.18%)	−2.63	0.65				
						$KL_{y}$(11.61%)	−0.35	0.02
$D_{z}$	13.52				3.30	$C_{L}$(10.56%)	0.23	0
						$C_{T}$(21.63%)	0.33	−0.02
		$KL_{x}$(49.20%)	−0.84	0.10		$KL_{x}$(32.89%)	−0.40	0
		$KL_{z}$(32.63%)	0.61	0.07		$KL_{z}$(23.58%)	0.34	0
$F_{x}$	47.18	$KL_{x}$(98.05%)	−24.70	1.15	43.14	$KL_{x}$(96.26%)	−25.78	−2.12
$F_{y}$	11.15	$KL_{y}$(81.95%)	−7.456	−1.65	16.72	$KL_{y}$(92.41%)	−9.05	0
$F_{z}$	63.48	$KL_{z}$(99.01%)	−26.83	2.14	30.08	$KL_{z}$(99.08%)	−11.85	1.13
$M_{x}$	1.00	$C_{T}$(65.94%)	−0.46	0	1.04	$C_{T}$(89.30%)	−0.48	0
		$KL_{z}$(27.19%)	−0.30	0.01				
$M_{y}$	5.72				2.43	$KL_{x}$(32.02%)	−0.78	0.05
		$KT_{y}$(94.78%)	−2.32	0.14		$KT_{y}$(59.65%)	−1.05	0.04
$M_{z}$	2.04	$C_{T}$(63.76%)	−0.85	0.03	2.88	$C_{T}$(95.16%)	−1.24	0.05
		$KL_{x}$(30.53%)	−0.60	0.05				

## Abbreviations

The following abbreviations are used in this manuscript:

DOE	Design of Experiments
ANOVA	Analysis of Variance
